# The complete mitochondrial genomes of three imperiled cyprinid fishes Bonytail (*Gila elegans*), Rio Grande Silvery Minnow (*Hybognathus amarus*) and Loach Minnow (*Tiaroga cobitis*)

**DOI:** 10.1080/23802359.2020.1774435

**Published:** 2020-06-08

**Authors:** Megan J. Osborne, Alexander C. Cameron, Brian P. Fitzgerald, Samuel A. McKitrick, Madison R. Paulk, Thomas F. Turner

**Affiliations:** Department of Biology and Museum of Southwestern Biology, University of New Mexico, Albuquerque, New Mexico, USA

**Keywords:** Bonytail, Rio Grande Silvery Minnow, Loach Minnow, mitogenomes, conservation genetics

## Abstract

*Gila elegans*, *Hybognathus amarus*, and *Tiaroga cobitis* (Family Cyprinidae, Order Cypriniformes) are endemic and endangered fishes in the southwestern United States. We present complete mitochondrial genomes for each species. Each mitochondrion consisted of 13 protein-coding genes, 2 ribosomal (rRNA) genes, 22 transfer RNA (tRNA) genes, and a single control region (D-loop), and gene order was consistent with other cyprinid fishes. Total genome lengths were 16,593 base pairs (bp) for *G*. *elegans*, 16,705 bp for *H*. *amarus*, and 16,802 for *T*. *cobitis.* The GC content in *G*. *elegans* and *H. amarus* was 44%, but higher in *T*. *cobitis* at 48%. Phylogenetic trees were generated to confirm relationships inferred via novel mitogenomes, and best-supported trees were consistent with previous research.

The vertebrate mitochondrial genome contains a highly conserved and well-understood gene composition and order, and yet can exhibit a rapid rate of sequence divergence between species and populations. These properties of the mitochondrial genome are useful for distinguishing and comparing recently diverged lineages (e.g. Ratnasingham and Hebert 2007). As such, mitochondrial genome studies have shed light on an array of ecological and evolutionary aspects of vertebrate life. Moreover, the mitochondrial genome is a valuable resource in conservation genetic research as it can be utilized for understanding changes in population dynamics and evolutionary history (Taguchi et al. 2015; Osborne et al. 2019) and in environmental DNA (eDNA) applications (e.g. Bronnenhuber and Wilson 2013; Dysthe et al. 2016). Fishes of the southwestern United States can benefit from comparative mtDNA research because many taxa have experienced precipitous declines and are subject to active management. Here, we assembled and annotated mitochondrial genomes for three western cyprinids: Bonytail (*Gila elegans*), Rio Grande Silvery Minnow (*Hybognathus amarus*), and Loach Minnow (*Tiaroga cobitis*). All three species are federally listed as endangered and have experienced major declines due to alterations to the natural hydrograph, habitat loss, and the introduction of non-native fishes (Sublette et al. 1990; Minckley and Marsh 2009). All three species have hatchery breeding programs, and have been stocked into natural habitats as part of recovery efforts.

Genomic DNA was isolated from caudal fin tissue from a single individual of each taxon. *Gila elegans* was collected from Southwestern Native Aquatic Resource and Recovery Center (Dexter, New Mexico, 33.194667, −104.350647) and *H*. *amarus* was collected from the Rio Grande, New Mexico (approximate locality: 34.213738, −106.885898). *Tiaroga cobitis* was collected from hatchery broodstock maintained at the Arizona Aquatic Research and Conservation Center that originated from the upper Gila River in New Mexico (approximate locality: 33.2284105, −108.255927). For all species, any remaining tissue and/or DNA isolates were deposited in the Museum of Southwestern Biology (MSB ACC2014-V.23, ACC1993-VIII:27 and ACC2018-X:30). Paired-end reads (150 bp) from a single sequencing lane (Illumina NextSeq 500; University of New Mexico) for each individual were quality trimmed via trimmomatic v. 0.36 (Bolger et al. 2014). Remaining paired reads were each baited and assembled using MITObim (Hahn et al. 2013) with the full mitogenomes of *Gila robusta* (Genbank Accession: NC008105.1), *Hybognathus nuchalis* (Genbank Accession: NC031567.1) and *Rhinichthys cataractae* (Genbank Accession: MG570448.1) used as bait sequences for *G*. *elegans*, *H. amarus*, and *T. cobitis*, respectively. Annotation of novel mitochondrial genomes was performed using the MitoFish pipeline (Iwasaki et al. 2013).

In all taxa, each mitochondrion consisted of 13 protein-coding genes, 2 rRNA genes, 22 tRNA genes, and a D-loop control region. Gene order was identical to other cyprinid fishes. Complete genome lengths were 16,593 for *G*. *elegans*, 16,705 bp for *H*. *amarus* and 16,802 for *T*. *cobitis*. Minor genome length variation between species was the result of variation in the D-loop and origin of replication (Brown et al. 1993). Length of D-loop regions were 915 bp for *G. elegans*, 1042 bp for *H. amarus,* and 1135 bp in *T*. *cobitis*. Nucleotide content in *G. elegans* was 29% A, 27% T, 26% C, 18% G, *H. amarus* nucleotide content was 28% A, 29% T, 26% C, 17% G and nucleotide content for *T*. *cobitis* was 25% A, 27% T, 27% C, 21% G. Novel mitogenomes were imported into Mega7 (Kumar et al. 2016) and aligned with complete mitochondrial genomes of nine other cyprinid species and rooted with two representatives of the family Catostomidae. A neighbor-joining tree was constructed using composite maximum likelihood distances and node support was determined from 1000 bootstrap replicates. Results of the phylogenetic analysis revealed groupings consisted with previous research (Figure 1; Schönhuth et al. 2018).

**Figure 1.**
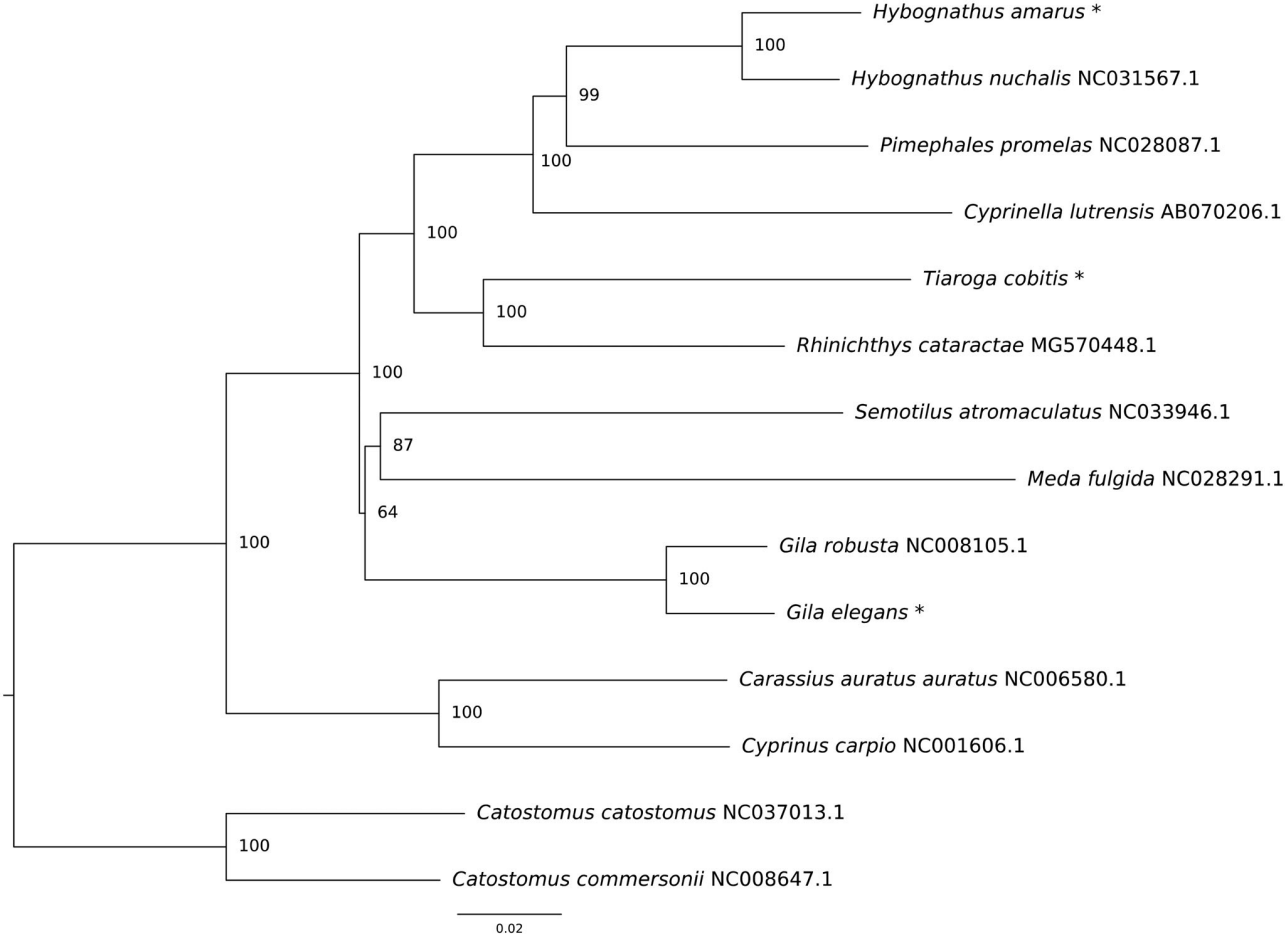
Optimal neighbor-joining phylogeny of fishes (Order Cypriniformes) constructed using complete mitochondrial genomes. Asterisks denote novel sequences. Node support values were generated via 1000 bootstrap replicates.

## Data Availability

The mitochondrial genome sequences reported here are available on Genbank using the following accession numbers: MT364325 (https://www.ncbi.nlm.nih.gov/nuccore/MT364325) MT364326 (https://www.ncbi.nlm.nih.gov/nuccore/MT364326) MT364327 (https://www.ncbi.nlm.nih.gov/nuccore/MT364327).
